# Design of Extractants for F-Block Elements in a Series of (2-(Diphenylphosphoryl)methoxyphenyl)diphenylphosphine Oxide Derivatives: Synthesis, Quantum-Chemical, and Extraction Studies

**DOI:** 10.3390/molecules26082217

**Published:** 2021-04-12

**Authors:** Alfiya Safiulina, Nataliya Borisova, Mikhail Grigoriev, Dmitriy Baulin, Vladimir Baulin, Aslan Tsivadze

**Affiliations:** 1 Joint Stock Company A. A. Bochvar High-Technology Research Institute of Inorganic Materials, ul. Rogova 5a, 123098 Moscow, Russia; amsafiulina@bochvar.ru; 2 Chemical Faculty, Moscow State University, Leninskie Gory 1, 119991 Moscow, Russia; borisova.nataliya@gmail.com; 3 Frumkin Institute of Physical Chemistry and Electrochemistry, Russian Academy of Sciences, Leninsky Prospect 31, Building 4, 119071 Moscow, Russia; mickgrig@mail.ru (M.G.); Atsiv43@mail.ru (A.T.); 4 Institute of Physiologically Active Substances, Russian Academy of Sciences, Severnyi Proezd 1, Chernogolovka, 142432 Moscow, Russia; mager1988@gmail.com

**Keywords:** phosphoryl-containing podands, synthesis, structure, quantum-chemical modelling, X-ray diffraction analysis extraction, preconcentration, separation of U(VI), Th(IV), REE(III), nitric acid

## Abstract

With the aim to find new efficient extractants for recovery of f-block elements from processing wastes of different origin, we have compared a series of phosphoryl-containing podands, including (2-(diphenylphosphorylmethoxy)phenyl)diphenylphosphine oxide **1** and its analogues **5**–**7**, where the ArP(O)Ph_2_ group of phosphine oxide type is replaced by phosphonic fragments. Quantum-chemical modelling of the structures of phosphoryl-containing podands **1** and **5**–**7** has been performed, which was later confirmed by the data of X-ray diffraction. The features of extraction of nitric acid, as well as U(VI), Th(IV), Nd(III), and Ho(III) with compounds **1** and **5**–**7** from nitric acid media into 1,2-dichloroethane have been studied. The compositions of extracted complexes have been determined.

## 1. Introduction

The production of concentrates and other products based on rare and rare earth metals (RM and REM) from ores and process materials results in a large amount of low-level wastes, whose radioactivity is caused mainly by the presence of uranium and thorium compounds. To provide environmental safety, environmental legislation requires their controlled long-term storage, in which expenditure considerably decreases the cost-effectiveness of RM and REM production.

Alternative strategy for radioactive waste handling is the selective separation of radionuclides and stable elements to decrease considerably the volume of radioactive waste and organize the cost-effective manufacture necessary for industry stable elements, for example, rare earth metals [1].

There are different processes for the selective recovery of components of low-level wastes (precipitative, sorption, chromatographic, etc.), but the liquid extraction method using organic compounds (extractants) is one of the most efficient and widely used in contemporary technology [2,3]. Organophosphorus compounds are widely used as extractants for the recovery of radionuclides [4]. However, at the present time, commercial neutral organophosphorus extractants belonging to phosphates, phosphanates, and phosphine oxide classes (tributyl phosphate, trioctylphosphine oxide, diphenyldibutylcarbamoylmethylphosphine oxide, etc.) are available. In addition, acidic phosphorus compounds such as di-(2-ethylhexyl)phosphoric acid do not always exhibit the necessary properties to solve the problems on the selective separation of complex solution components [5,6,7], therefore the synthesis and study of extraction properties of new organic compounds is an urgent task [8,9,10,11,12,13].

Podands, which are synthetically feasible acyclic analogues of crown ethers, are a very promising class of compounds for metal extraction. Since the phosphoryl group is easily polarized and has a great coordination ability, phosphoryl podands are of considerable scientific and practical interest. Moreover, an advantage of phosphoryl podands is the possibility to change the coordination properties of the phosphoryl group in a wide range by varying substituents at the phosphorus atom [13,14]. Furthermore, the methods of organophosphorus chemistry allow constructing a wide range of phosphoryl-containing podands of different conformational rigidities and diverse number of coordination centers, which opens wide possibilities for the targeted modification of their extraction properties [15,16,17,18]. A systematic study of the extraction properties of polydentate organic compounds is necessary to find new efficient extractants for elements important for the industry [19,20].

Among the studied phosphoryl podands, the neutral derivatives of (2-(diphenylphosphorylmethoxy)phenyl)diphenyl)phosphine oxide 1 show a high extraction ability towards Am(III), U(IV), Th(IV), and rare-earth elements (REE(III)) [21,22,23,24,25,26]. The influence of substituent nature at the phosphoryl group on the extraction ability and selectivity towards U(IV) and Th(IV) was studied in the works [25,26]. It was established that 3 is a very efficient extractant for lanthanides [26].

Extraction of REEs with phosphoryl podand **4** solutions in 1,1,7-trihydrododecafluoroheptanol from nitric acid solutions was studied. It was shown that an increase in HNO_3_ concentration above 1 M abruptly increases the distribution ratios of REEs up to 5.5 for the yttrium subgroup elements at the concentration of 6 M. All things being equal, the yttrium group щa REEs is better extracted than the cerium group of REEs. In both groups, distribution ratios increase gradually with the element number. Using the equilibrium shift method, it was shown that the M:L ratio in extracted complexes is 1:2 regardless of the REE nature. Using the X-ray diffraction analysis, the structure of neodymium complex was established. Its single crystals were grown from an extraction solution [27]. The same results were obtained for the erbium complex [28]. It was established that the replacement of CH_2_P(O)Ph_2_ group in dioxide **1** molecule by –C(O)NAlk_2_ alkylamide group significantly affects the extraction of REE(III) and Sc(III) ions [29].

On the other hand, it is known that phosphoryl podand derivatives of phosphonic acids are also efficient extractants for f-block elements [30,31,32].

**Figure 1:**
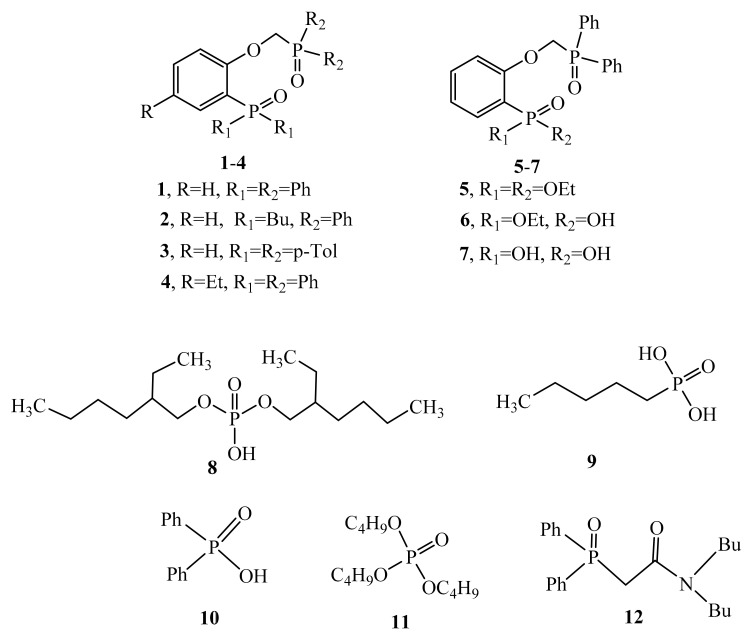
Chemical structures of the studied compounds.

In this work, we compared the extraction ability of (2-(diphenylphosphorylmethoxy)phenyl)diphenylphosphine oxide **1** and its analogues **5–7** in which the ArP(O)Ph_2_ group of phosphine oxide type was replaced by a phosphonic fragment (Figure 1) with the aim to find new efficient extractants. A quantum chemical modelling for the structures of compounds **1** and **5**–**7** was conducted. Next, the models were confirmed by the results of X-ray diffraction study. Modelling for the structure of complexes of the studied phosphoryl podands with thorium, uranium, and neodymium nitrates was also conducted. The features of extraction of nitric acid and U(VI), Th(IV), Nd(III), as well as Ho(III) from nitric acid solutions into 1,2-dichloroethane were studied. The extraction ability of compounds **1** and **5**–**7** towards U(VI), Th(IV), Nd(III), and Ho(III) was compared with reference acidic extractants such as di-(2-ethylhexyl) phosphoric acid (**8**), amylphosphonic (**9**), and diphenylphosphinic acids (**10**), as well as neutral extractants (tributylphosphate (**11**) and N,N-dibutyl-2-(diphenylphosphoryl)acetamide (**12**)).

## 2. Experimental

The melting points were determined in open capillary tubes on a melting point apparatus. The TLC of individual compounds and reaction mixtures was carried out on Silufol plates (3–7% chloroform–isopropanol as an eluent).

^1^H and ^31^P NMR spectra were recorded on a BrukerCXP-200 spectrometer using TMS as an internal and 85% H_3_PO_4_ as an external reference. 

**Diethyl (2-((diphenylphosphoryl)methoxy)phenylphosphonate 5.** A 55% suspension of sodium hydride (0.68 g, 15.4 mmol) in liquid paraffin was added to 3.55 g (15.4 mmol) of the solution of diethyl(2-hydroxyphenyl)phosphonate in 45 mL of dry dioxane. The mixture was heated to the boiling point and stirred for 15 min. Next, 5.71 g (15.4 mL) of (diphenylphosphoryl)methyl 4-methylbenzenesulfonate was added. The mixture was stirred for 8 h and dioxane was removed under reduced pressure. One hundred milliliters of water was added to the residue, it was acidified with concentrated HCl to pH = 1, and the mixture was extracted with chloroform (3 × 30 mL). The extract was washed with water (3 × 25 mL) and the solvent was concentrated under reduced pressure. Hexane (50 mL) was added to the residue and the sediment 5 was filtered out. According to the data of thin layer chromatography and ^31^P NMR spectra, the obtained sample 5 contains up to 15–20% impurities (starting compounds diethyl (2-hydroxyphenyl) phosphonate and (diphenylphosphoryl) methyl 4-methylbenzenesulfonate and, possibly, their hydrolysis products). An analyte sample of 5 was obtained by preparative column chromatography with L-type silica gel. The eluent was chloroform and i-PrOH–chloroform mixture (10:1). The yield of compound **5** was 4.85 g, 71%, mp = 91–93 °C (benzene-hexane). Found, %: C 61.91, 62.07; H 5.55, 5.78; P 13.74, 13.87. For C_23_H_26_O_5_P_2_ anal. calcd., %: C 62.16; H 5.90; P 13.94. ^1^H NMR spectrum (200 MHz, CDCl_3_, δ, ppm): 1.16 t (^3^Jн-н = 7.12 Hz, 6H, 2OCH_2_CH_3_); 3.94 m (4H, 2OCH_2_CH_3_); 4.85 d(^2^Jн-p = 7.4 Hz, 2H, CH_2_P(O)Ph_2_); 7.08 m (2H, Ar-H); 7.53 m (7H, Ar-H); 7.85 m (1H, Ar-H); 8.03 m (4H, Ar-H). ^31^P NMR spectrum (81 MHz, CDCl_3_, δ, ppm): 17.34; 26.08. ^13^C NMR spectrum (50 MHz, CDCl_3_, δ, ppm): 6.44; 62.16; 67.24; 112.68; 117.37; 121.93; 128.77; 130.45; 132.00; 132.58; 134.71; 135.59; 160.31.

**Ethyl (2-((diphenylphosphoryl)methoxy]phenyl)phosphonate 6.** A solution containing 4.44 g (10 mmol) of compound **5**, 2.00 g (50 mmol) NaOH in 30 mL of 50% ethyl alcohol was heated under reflux for 8 h, cooled, acidified with concentrated HCl to pH = 1 and extracted with chloroform (3 × 30 mL). The extract was washed with water (3 × 25 mL) and concentrated under reduced pressure. Dry diethyl ether (20 mL) was added to the residue and the sediment was filtered out and recrystallized from isopropyl alcohol. The yield of compound **6** was 3.62 g, 87%, mp = 152–154 °C (i-PrOH). Found, %: C 60.42, 60.56; H 5.24, 5.19; P 14.53, 14.65. For C_21_H_22_O_5_P_2_ anal. calcd., %: C 60.58; H 5.33; P 14.88. ^1^H NMR spectrum (200 MHz, CDCl_3_, δ, ppm): 1.15 t (^3^Jн-н = 6.8 Hz, 3H, OCH_2_CH_3_); 4.00 m (2H, OCH_2_CH_3_); 4.89 d (^2^Jн-p = 2.75 Hz, 2H, CH_2_P(O)Ph_2_); 7.05 m (2H, Ar-H); 7.59 m (7H, Ar-H); 7.95 m (5H, Ar-H); 10.93 s (1H, P(O)-OH). ^31^P NMR spectrum (81 MHz, CDCl_3_, δ, ppm): 15.19; 30.91.^13^C NMR spectrum (50 MHz, CDCl_3_, δ, ppm): 16.45; 62.38; 68.11; 112.68; 117.37; 121.93; 128.77; 133.45; 131.99; 132.58; 134.68; 135.59, 160.31.

**2-((Diphenylphosphoryl)methoxy)phenylphosphonic acid 7.** Anhydrous sodium bromide (4.12 g, 40.0 mmol) and 4.32 g (40 mL) of trimethylchlorosilane were added to a solution of 5.31 g (10.5 mmol) of compound **5** in 30 mL of dry acetonitrile. The reaction mixture was heated under reflux for 6 h, cooled, the precipitate was filtered off and the filtrate was concentrated under reduced pressure. Water (20 mL) was added to the residue and the sediment was filtered out and recrystallized from ethyl alcohol. The yield of compound **7** was 3.25 g, 84%, mp = 268–270 °C (ethanol). Found, %: C 58.64, 58.71; H 4.43, 4.57; P 15.80, 15.67. For C_19_H_18_O_5_P_2_ anal. calcd., %: C 58.77; H 4.67; P 15.95. ^1^H NMR spectrum (200 MHz, DMSO-d_6_, δ, ppm): 3.80.00 bs (2H, 2P(O)(OH)_2_ + H_2_O from DMSO-d_6_); 5.00 d (^2^Jн-p = 5.9 Hz, 2H, CH_2_P(O)Ph_2_); 7.06 m (1H, Ar-H); 7.22 m (1H, Ar-H); 7.57 m (8H, Ar-H); 7.95 m (4H, Ar-H). ^31^P NMR spectrum (81 MHz, DMSO-d_6_, δ, ppm): 10.94; 27.63.^13^C NMR spectrum (50 MHz, DMSO-d_6_, δ, ppm): 68.08; 113.96; 120.39; 122.13; 122.18; 129.16; 124.44; 131.80; 133.88; 134.01; 160.10.

Quantum chemical calculations were performed using an MVS-50K supercomputer of the Joint SuperComputer Center of the Russian Academy of Sciences (www.jscc.ru). All the calculations were made using the Priroda Software [33,34] (PBEO functional [35]. For all the systems, the expansion of electronic density in auxiliary basis sets was used. The geometry of all the compounds was optimized without symmetry limitations. The analysis of vibrational spectra was used for identifying stationary points. The energy chacteristics were calculated with adjustment for the vibrational zero-point level. The TZ type basis set was applied to study the conformational behavior of all the atoms [33]. For all studied conformers, the values of the relative energy and the Gibbs free energy of formation of conformers were obtained as the difference of the relevant values for one compound. 

In order to study structures of supposed complexes with f-block elements, the calculations were made using the cc-pVDZ basis [33]. The original molecule geometries were based on X-ray diffraction data.

### 2.1. X-ray Diffraction Analysis

Colorless translucent crystals of compounds **6** and **7** which can be subjected to the X-ray structural analysis were obtained by isothermal evaporation of the compounds in 1,2-dichloroethane at room temperature. X-ray diffraction experiments were conducted using an automatic 4-circle Bruker KAPPA APEX II diffractometer with an area detector [36] (graphite monochromated MoKα radiation) at 100 K. The parameters of the unit cells were refined for the whole dataset. The structures were solved by the direct method (SHELXS97 [37]) and refined by the full-matrix least-squares technique (SHELXL-2018/3 [38]) on F_2_ for all the data with anisotropic approximation for all the non-hydrogen atoms. H atoms of CH, CH_2_, and CH_3_ groups are located in geometrically calculated positions, with UH = 1.2 Uequ(C) for CH and CH_2_ groups and UH = 1.5 Uequ(C) for CH_3_. H atoms of OH groups are located by difference Fourier analysis and refined with individual temperature parameters and O–H distance limitations. The details of X-ray structural analysis and the main crystallographic data are given in Table 1. The atom coordinates were deposited with the Cambridge Crystallographic Data Centre, CCDC Deposition Numbers 2003557–2003558.

### 2.2. Study of Metal Extraction

The solutions were prepared in bidistilled water: 1,2-dichloroethane (reagent grade), Arsenazo (analytical grade), HNO_3_ (high purity grade), State standard sample 8363-2003 triuranium octoxide attested for content of uranium at 84.784 ± 0,016%, Th(NO_3_)_4_·4H_2_O (reagent grade), La(NO_3_)_3_·6H_2_O (reagent grade), Nd(NO_3_)_3_·6H_2_O (reagent grade), Ho(NO_3_)_3_·6H_2_O (reagent grade). The solutions of metal nitrates were prepared using the weight-in-volume method. Nitrate solutions of the studied elements were prepared by dissolving weights of corresponding nitrates in 0.01 mol/L solution of HNO_3_. The concentration of metal nitrates (0.1 mmol/L) was ascertained spectrophotometrically on a Thermo Scientific Gallery spectrophotometer. 

**Spectrophotometric method for the determination of actinides and lanthanides with arsenazo III** [39,40]**.** In a 25 mL flask, 1 mL of 0.1% arsenazo III solution, 1 mL of 0.5 mol/L HNO_3_, 0.5 mL of the volume of the metal sample were added and diluted to 25 mL. Photometric measurements were carried out at a wavelength of 650 nm in a 1 cm cuvette. In addition, 0.1% Arsenazo III solution was prepared in bidistilled water. Calibration curves were obtained in a similar way using standard solutions of actinides and lanthanides in a volume of 0.5 to 5 mL. The concentration of HNO_3_ solutions was determined by potentiometric titration of 0.1 mol/L NaOH on an S470 SevenExcellence™ pH/conductivity meter (Mettler Toledo) with the precision of ±0.01 pH. The electrode pair was calibrated with standard buffer solutions with pH = 1.68, 4.01, and 9.21 (Mettler Toledo) (values at 20 °C). The concentration of NaOH was ascertained by potentiometric titration with 0.1 mol/L of HCl (Fixanal).

The study of extraction of metal cations was conducted as follows. The nitric acid solution of 1.5 mL was added into a test tube with a stopper, its concentration varied from 0.053 to 5.15 mol/L, as well as 0.5 mL of 0.1 mmol/L metal nitrate solution, 2 mL of 0.01 mol/L solution of ligand in 1,2-dichloroethane. The phases were mixed for 20 min in a rotator (Multi RS-60, BioSan, 80 rpm). The time of reaching the equilibrium was checked by increasing the time of contact of phases up to 120 min with no change of distribution ratios. Phase stratification was performed by centrifugation. After the separation of the phases, the concentration of metal cations in the aqueous phase was determined spectrophotometrically. Not fewer than three independent tests were made for each concentration. With *n* = 3 and confidence level of *p* = 0.95, the total margin of error is δ = 0.33. Therefore, the confidence interval for the spectrophotometrically determined metal concentrations is 3.3 × 10^−6^ mol/L. All the experiments were conducted at 20 ± 1 °C. The extraction distribution ratios (D = [M]org/[M]aq) were determined with constant extractant concentrations (1 mmol/L in 1,2-dichloroethane) and initial experimental metal concentrations (0.025 mmol/L in the aqueous phase).

The concentration of nitric acid during extraction was determined by potentiometric titration. At least five independent experiments were conducted for each concentration. The accuracy of the results when studying the extraction of nitric acid was ~20% allowing for not excluded and random components.

## 3. Results and Discussion

**Figure 2:**
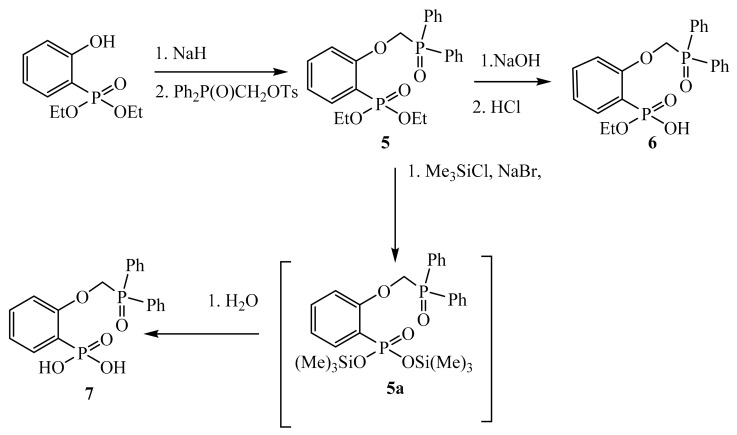
Scheme of synthesis of compounds **5**–**7**.

Compound **1** was obtained by the method described earlier [21]. Compound **5** was synthesized by alkylation of diethyl(2-hydroxyphenyl)phosphonate with (diphenylphosphoryl)methyl 4-methylbenzenesulfonate in the presence of sodium hydride in boiling dioxane. Semi-ether **4** was obtained by alkaline hydrolysis in a NaOH water-alcohol solution. Bis(trimethylsilyl) ether **5a** obtained through the reaction of **5** with trimethylbromosilane, which is formed by the reaction of trimethylchlorosilane with sodium bromide in boiling acetonitrile, was without isolation hydrolyzed with water to form phosphonic acid **7** (Figure 2).

### 3.1. X-ray Crystallography of Compounds **6** and **7**

**Figure 3:**
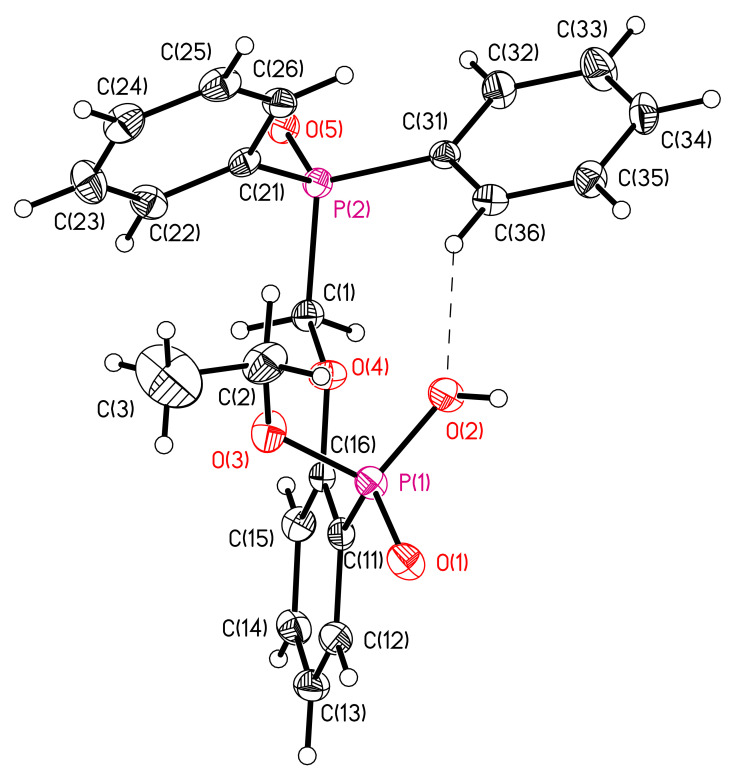
Molecular structure **6**. Bond lengths (Å): P(1)-O(1) 1.4827(18), P(1)-O(2) 1.565(2), P(1)-O(3) 1.5923(19), P(1)-C(11) 1.798(3), P(2)-O(5) 1.5111(17), P(2)-C(1) 1.832(3), P(2)-C(21) 1.816(3), P(2)-C(31) 1.801(2). The ellipsoids of temperature displacements are presented with a probability of 50%. H atoms are given as spheres of arbitrary radius. The H-bond is shown by a dashed line.

**Figure 4:**
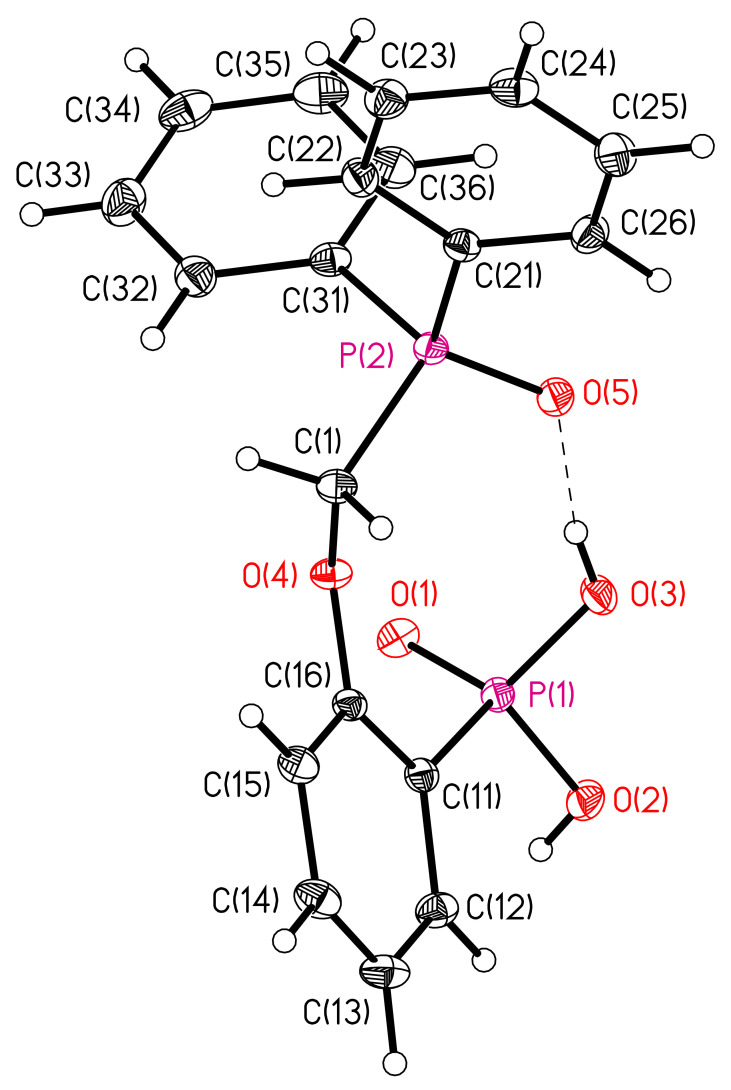
Molecular structure **7**. Bond lengths (Å): P(1)-O(1) 1.4969(9), P(1)-O(2) 1.5550(9), P(1)-O(3) 1.5481(9), P(1)-C(11) 1.8002(11), P(2)-O(5) 1.5021(9), P(2)-C(1) 1.8219(12), P(2)-C(21) 1.7901(11), P(2)-C(31) 1.7926(12). The ellipsoids of temperature displacements are presented with a probability of 50%. H atoms are given as spheres of arbitrary radius. The H-bond is shown by a dashed line.

**Figure 5:**
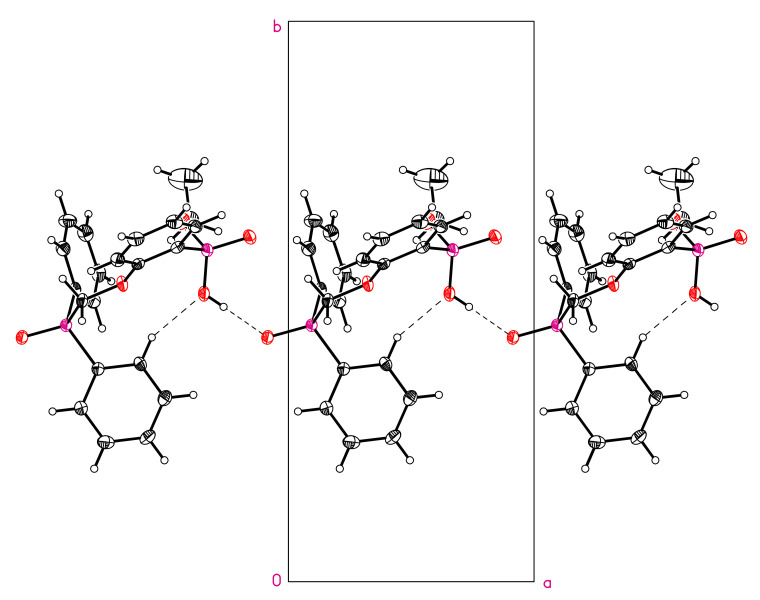
Chain formed by H-bonds in structure **6**. Projection in the [001] direction.

**Figure 6:**
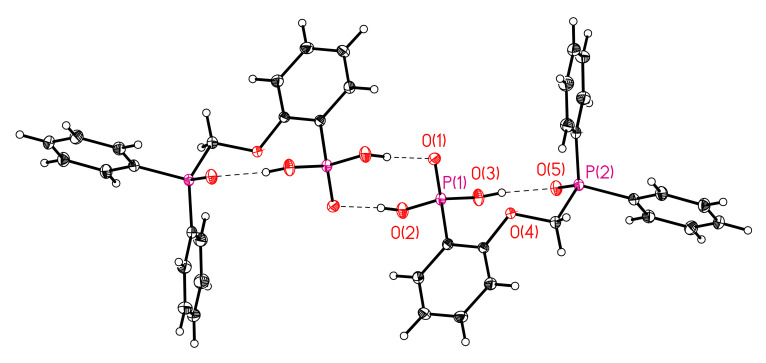
Centrosymmetric dimer in structure **7**.

The structures of compounds **6** and **7** were established by X-ray diffraction. The geometry of molecules and atom numbering schemes are shown in Figure 3 and Figure 4. The interatomic distances and bond angles around the P atoms are close in both structures (Table S1). Both structures include intermolecular H-bonds (Figure 3 and Figure 4, Table 2). There is a weak C–H…O bond in compound **6** and a strong O–H…O bond in compound **7**. The strong intermolecular H-bonds of O–H…O type (Table 2) produce chains in the direction [100] in structure **6** (Figure 5) and centrosymmetric dimers in structure **7** (Figure 6).

**Figure 7:**
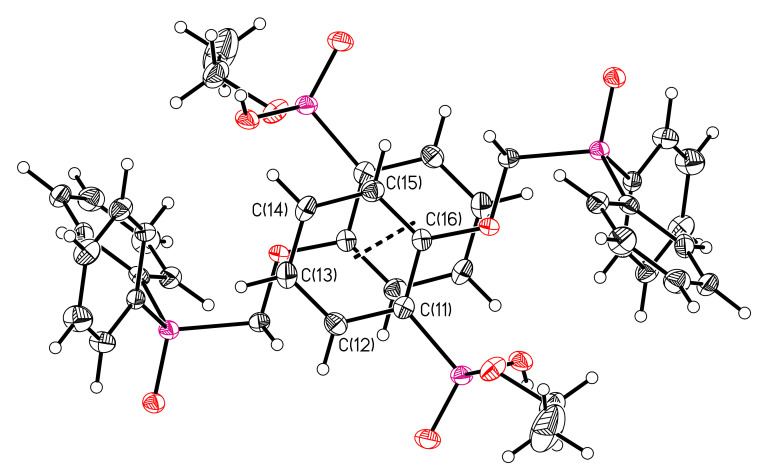
Pi stacking interaction in structure **6**.

In compound **6,** the molecules of neighboring chains are bound by pi stacking interactions between phenyl rings C(11)C(12)C(13)C(14)C(15)C(16) (Figure 7). The distance between the ring planes is 3.657 Å, the distance between the ring centroids is 3.910 Å and the shift is 1.384 Å. 

Experimental study of nitric acid extraction. Quantum chemical modelling of protonated complexes.

**Figure 8:**
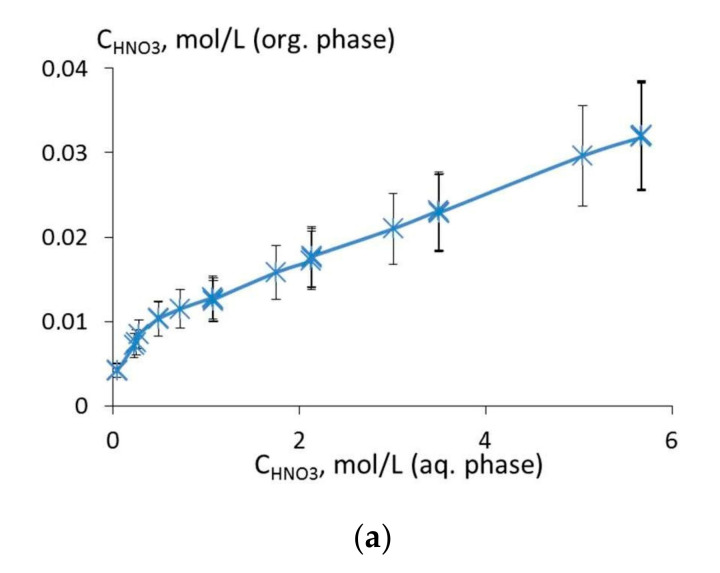

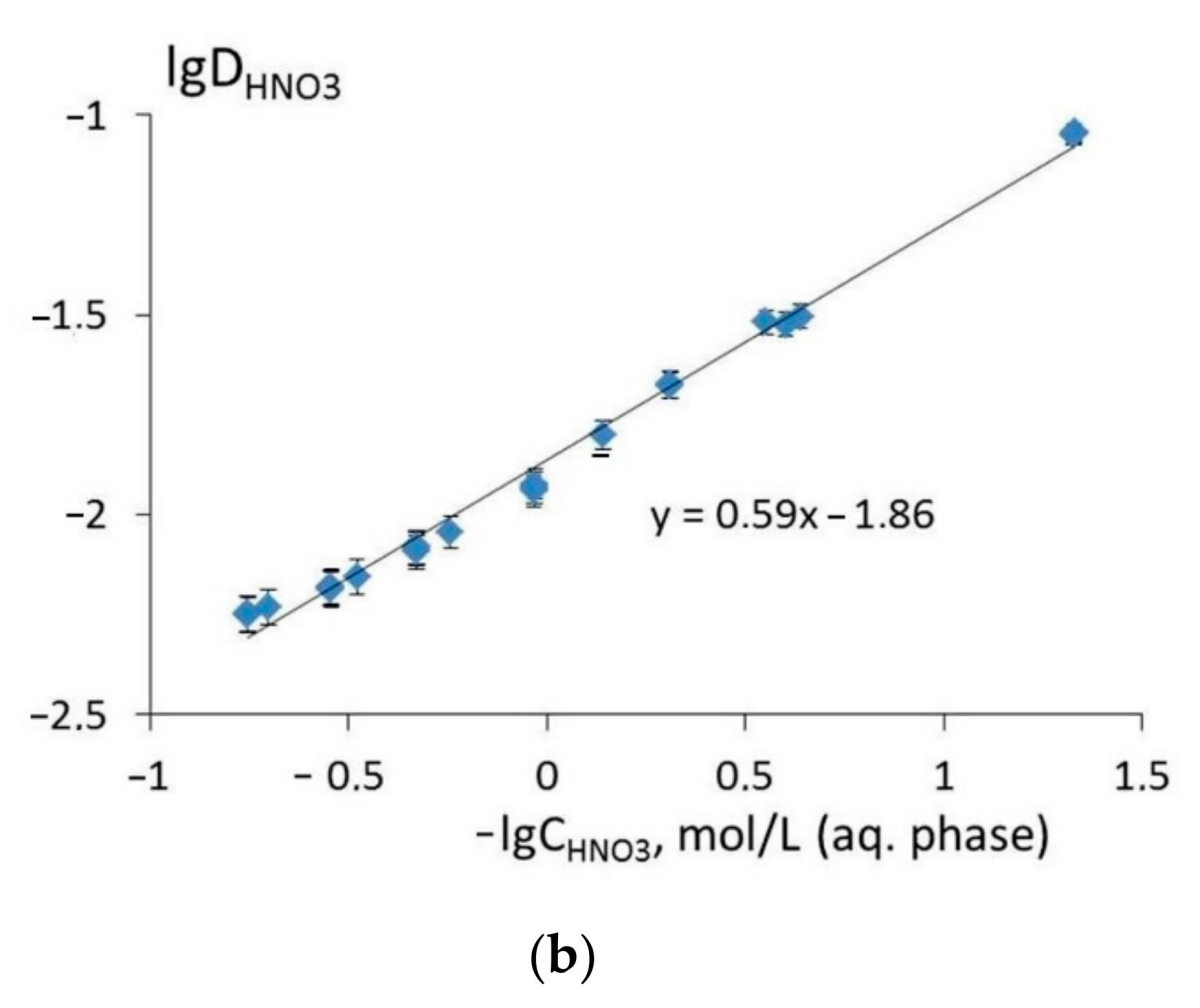
The extraction of nitric acid with a 0.01 mol/L solution of ligand **6** in 1,2-dichloroethane: (**a**) The extraction isotherm at 20 °C; (**b**) logarithmic dependence of the distribution ratio of nitric acid on its equilibrium concentration in aqueous phase.

Since the extraction of REEs from nitric acid media competes with the extractant interaction with nitric acid, which decreases free extractant concentration, we studied the extraction of nitric acid with a 0.01 mol/L solution of ligand **6** in 1,2-dichloroethane (Figure 8a,b). The isotherm of HNO_3_ extraction with a 0.01 mol/L solution of reagent **6** in 1,2-dichloroethane does not reach a saturation plateau within the studied range of HNO_3_ concentrations (Figure 8a). By the equilibrium-shift method, we showed that the stoichiometric ratio of ligand:HNO_3_ = 1:2, which means that one reagent molecule extracts two molecules of nitric acid (Figure 8b).

In order to clarify the protonation positions of compound **6**, the quantum chemical modelling of structures of organic reagents was conducted as calculations of densify functional of chemical shifts. In the relaxed structures of compounds **1**, **5**, **6,** and **7**, a negative charge is located on the oxygen atoms of two phosphoryl groups (Table 3). For neutral reagents **1** and **5**, charges on the oxygen atoms of the P=O groups are close despite the different structure of substituents at the phosphor atom (R=Ph for compound **1** and R=OEt for compound **5**). For acid reagents **6** and **7**, the greatest charge is carried by the oxygen atom of O=P(R_2_)CH_2_ group, while a smaller negative charge is located at the oxygen atom of the phosphoryl group of the phosphonic fragment. It is important to pay attention to the considerable negative charge at the oxygen atom in the O=P(R_2_)CH_2_ fragment for extractants **6** and **7** as compared to the charge of analogous fragments of **1** and **5**. In reagents **6** and **7**, there is a strong intramolecular hydrogen bond between the oxygen atom of O=P(R_2_)CH_2_ group and the oxygen atom of the phosphonic acid residue (Table 3). Formation of strong hydrogen bonds between the atoms of two O=P groups and nitric acid molecules can be suggested, which explains the observed stoichiometry of extraction of nitric acid by reagent **6**. As a result, the formation of a twice protonated reagent is supposed, in which one proton binds to the phosphine oxide group, as in neutral monodentate organophosphorus compounds, while the other proton protonates the oxygen atom of the O=P group of phosphonic acid, as in derivatives of organophosphorus acids, for instance, **8** [8].

According to the data of quantum chemical modelling, the studied reagents can be categorized into two groups in terms of the relative distribution of charges on oxygen atoms of P=O groups: The charges on oxygen atoms of P=O groups in compounds **1** and **5** are close to each other, so one cannot single out a more basic oxygen atom, while the charge on the oxygen atom of O=P(R_2_)CH_2_ group in compounds **6** and **7** exceeds considerably not only that of the oxygen atom of P=O group of phosphonic acid, but also analogous charges in reagents **1** and **5**. 

### 3.2. Extraction of Th(IV), Nd(III), U(VI), and Ho(III) and Quantum Chemical Modelling for the Structure of Extracted Complexes 

**Figure 9:**
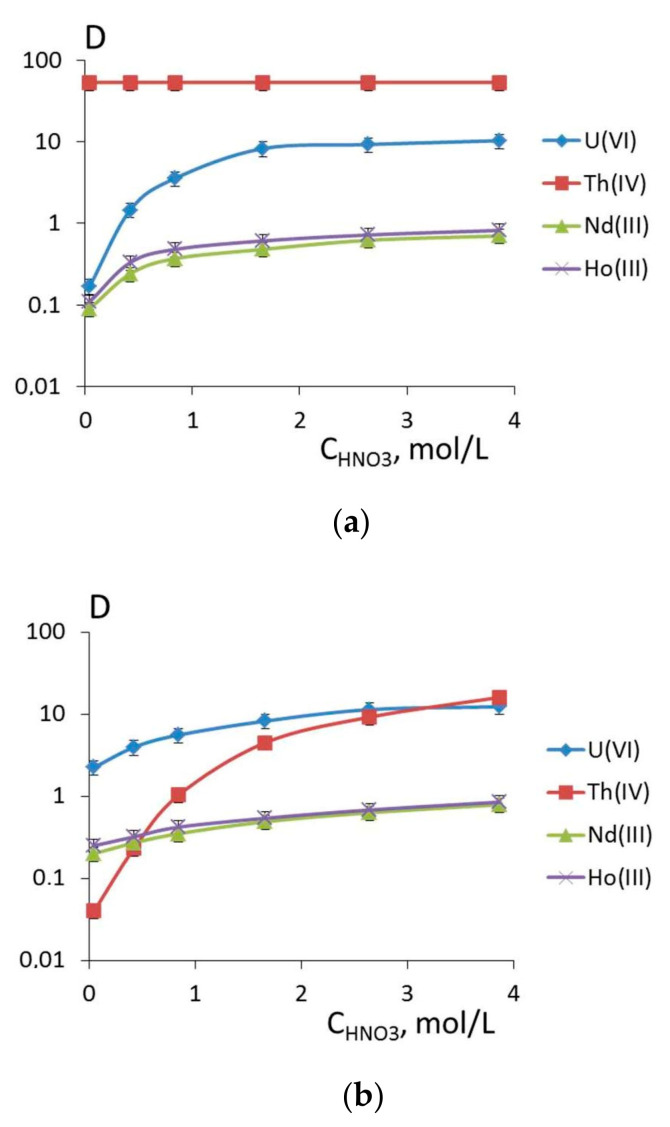
Dependence of logarithm of distribution ratios of a series of f-block elements on the nitric acid concentration upon extraction by 0.01 mol/L solutions of ligands (**a**) **1** and (**b**) **5** in 1,2-dichloroethane.

The results of experimental study for U(VI), Th(IV), Nd(III), and Ho(III) extraction by neutral reagents **1** and **5** depending on the nitric acid concentration are given in Figure 9.

The revealed features of U(VI), Nd(III), and Ho(III) extraction are described by the classical dependence of extraction with saturation. The extraction maximum for the extraction of the metals is observed for the nitric acid concentration of 1.66 M for reagent **1** and 2.64 M for reagent **5**. It should be noted that the extraction ability of these reagents towards U(VI) is considerably higher (D_U_~10 at C_HNO3_ > 2 mol/L) than that towards lanthanides (D_Nd_~0.7, D_Ho_ ~ 0.8 at C_HNO3_ > 2 mol/L).

There is a marked difference in the extraction of thorium ions with reagents **1** and **5**. The distribution ratios of Th(IV) extracted by the 0.01 M solution of reagent **5** increase monotonously with the HNO_3_ concentration in the initial solution and D_Th(IV)_ reaches 16 at C_HNO3_ = 3.83 mol/L. Under the same experimental conditions, reagent **1** extracts Th(IV) practically completely (D_Th_ ~53) within the whole range of nitric acid concentrations.

In order to explain this fact, we conducted quantum chemical modelling for the structure of Th(IV) complexes with reagents **1** and **5** relying on the earlier established fact that the stoichiometry of extracted complexes with thorium ions corresponds to the formation of ThL and ThL_2_ species [16] (Table 4).

**Figure 10:**
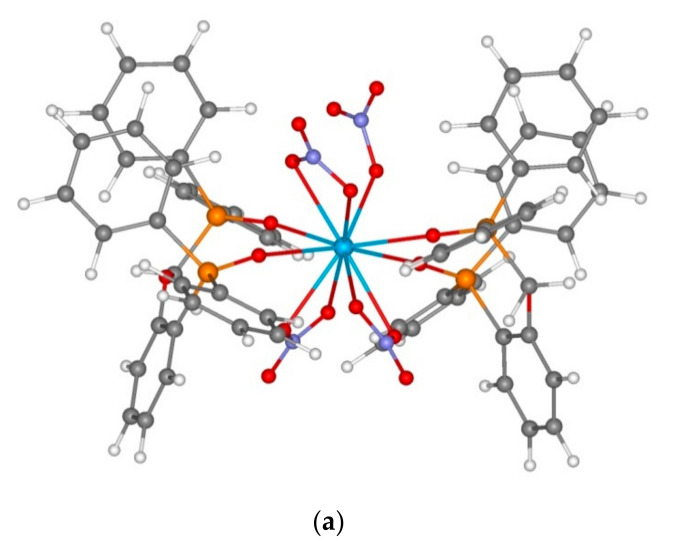

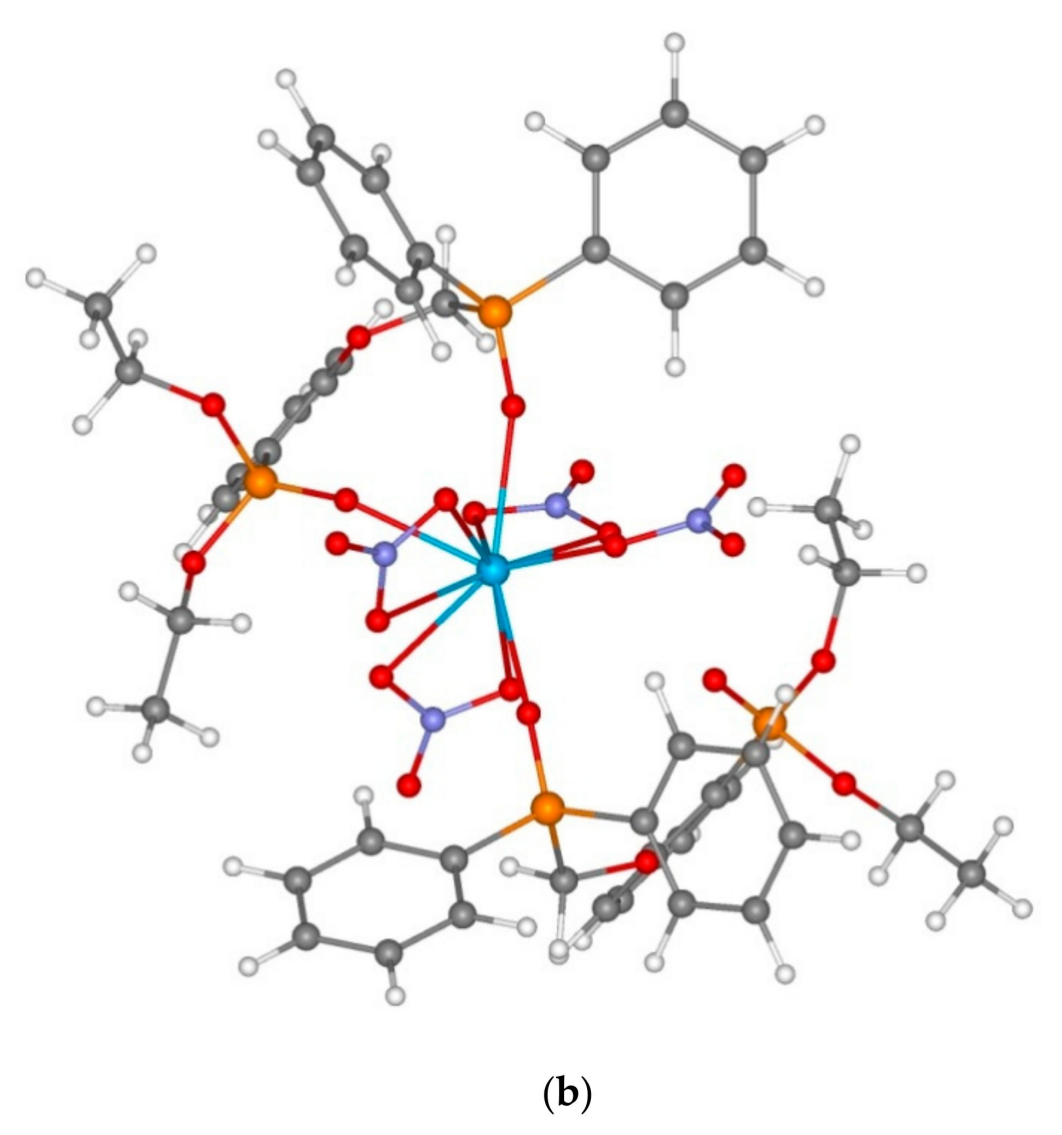
Structures of model complexes **1_2_Th(NO_3_)_4_** (**a**) and **5_2_Th(NO_3_)_4_** (**b**).

All the model complexes have negative complexation energies, which confirms the general possibility of complex formation. The structure of the complexes insignificantly depends on the character of the ligand and the ligand coordination type is mainly determined by the complex composition. Model monometal complexes of ligands **1** and **5** of composition LTh(NO_3_)_3_^3+^ are thorium complexes with the coordination number equal to 8, which contain nitrate anions with the bidentate-bridging coordination as well as a ligand with the bidentate coordination. It is noteworthy that the ether oxygen atom of ArOCH_2_P(O)Ph_2_ bridge is outside the binding distance of the metal ion (over 4 Å for all the studied ligand types and complex compositions). From the provided data (Table 4) it is evident that the complexation of thorium with diphosphine dioxide **1** is more preferable than that with neutral phosphonate **5**. This is proved by a higher energy of complexation for LTh(NO_3_)_3_^3+^ cation complex as well as by considerably shorter distances in the oxygen-metal bonds of the phosphine oxide part of the molecule than that of the phosphonate part (Table 4, nos. 1 and 4). A study of the influence of the complex composition on the values of complexation energies was conducted using the well-studied ligand **1** (Table 4, nos. 1 and 5). Introduction of the second reagent molecule into the cation complex composition was found to result in a smaller energy gain per one ligand in the complex. As for the formation of neutral complex L_2_Th(NO_3_)_4_, it is even less advantageous in energy since the complexation energy (Equation (2)) is only 60.40 kcal/mol (Table 4, no. 6). For neutral phosphonate **5,** this energy is even lower, only 56.12 kcal/mol (Table 4, no. 9). It is also noteworthy that in the case of neutral phosphonate **5,** the introduction of the second ligand molecule into the complex results in its monodentate coordination at the phosphine oxide fragment, as compared with the bidentate coordination of monoligand complexes and the complex of reagent **1** (Figure 10). Therefore, a greater affinity of phosphine oxide **1** to thorium ions can account for the large distribution ratios of the metal during its extraction and the lack of dependence on the nitric acid concentrations within the chosen range, as shown in Figure 9.

Th(NO_3_)_3_^+^ + nL = L_n_Th(NO_3_)_3_^+^ (1)

Th(NO_3_)_4_ + 2L = L_2_Th(NO_3_)_4_ (2)

**Figure 11:**
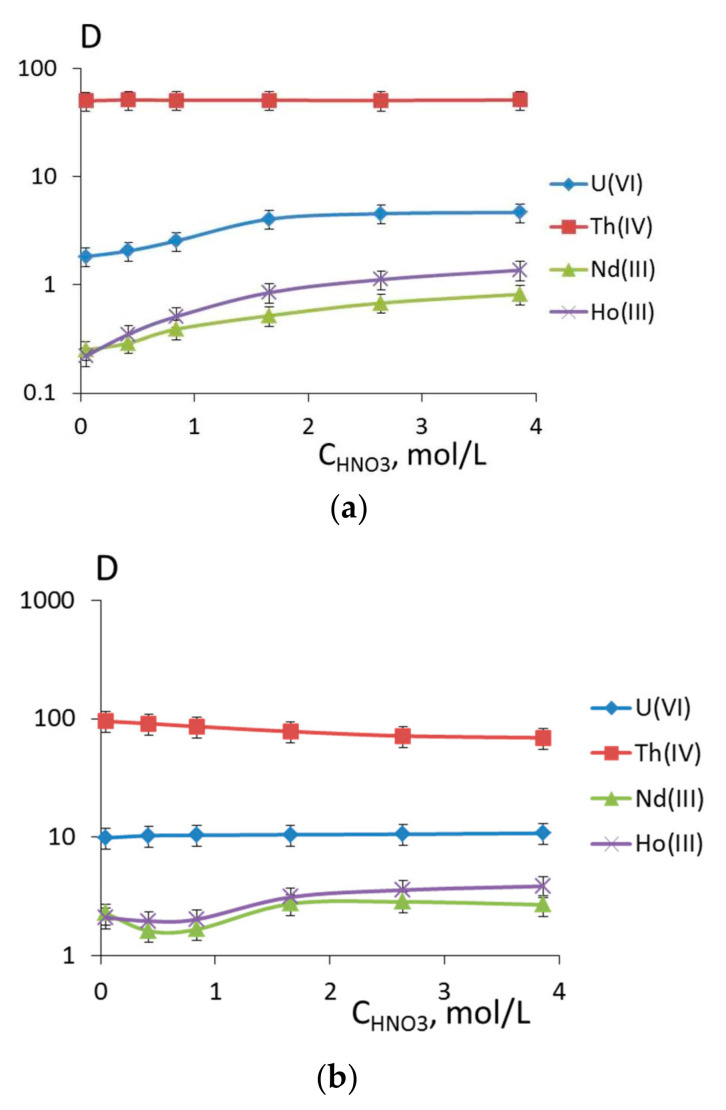
Dependence of the logarithm of distribution ratios of metals on the nitric acid concentration during extraction with 0.01 mol/L solutions of ligands (**a**) **6** and (**b**) **7** in 1,2-dichloroethane.

Another group of reagents is dibasic acids **6** and **7**. For them, the extraction of ions of f-block elements depending on the nitric acid concentration was also studied (Figure 11). The extraction of lanthanides with the reagents depends on the basicity of acid: The extraction of lanthanides with the monobasic acid **6** follows the classical curve with saturation (the maximal extraction is attained above the level of 2.64 mol/L of nitric acid); while the dependence of distribution ratios of neodymium and holmium for the dibasic acid is analogous to that observed for organophosphorus acidic extractants, for instance, **8** [8]. Likewise, the dependence of uranium extraction with monobasic acid **6** is in the middle of the curves for reagents **5** and **7**.

The extraction of thorium does not depend on the acidity of the medium, as for reagent **1**.

If uranium(VI) is extracted with a 0.01 mol/L solution of extractant **6** in 1,2-dichloroethane, the distribution ratios are lower than those obtained for extractants **1**, **7,** and **5**. The distribution ratios of neodymium and holmium for dibasic acid **7** are higher than for monobasic acid **6** or bidentate reagents **1** and **5**. 

**Figure 12:**
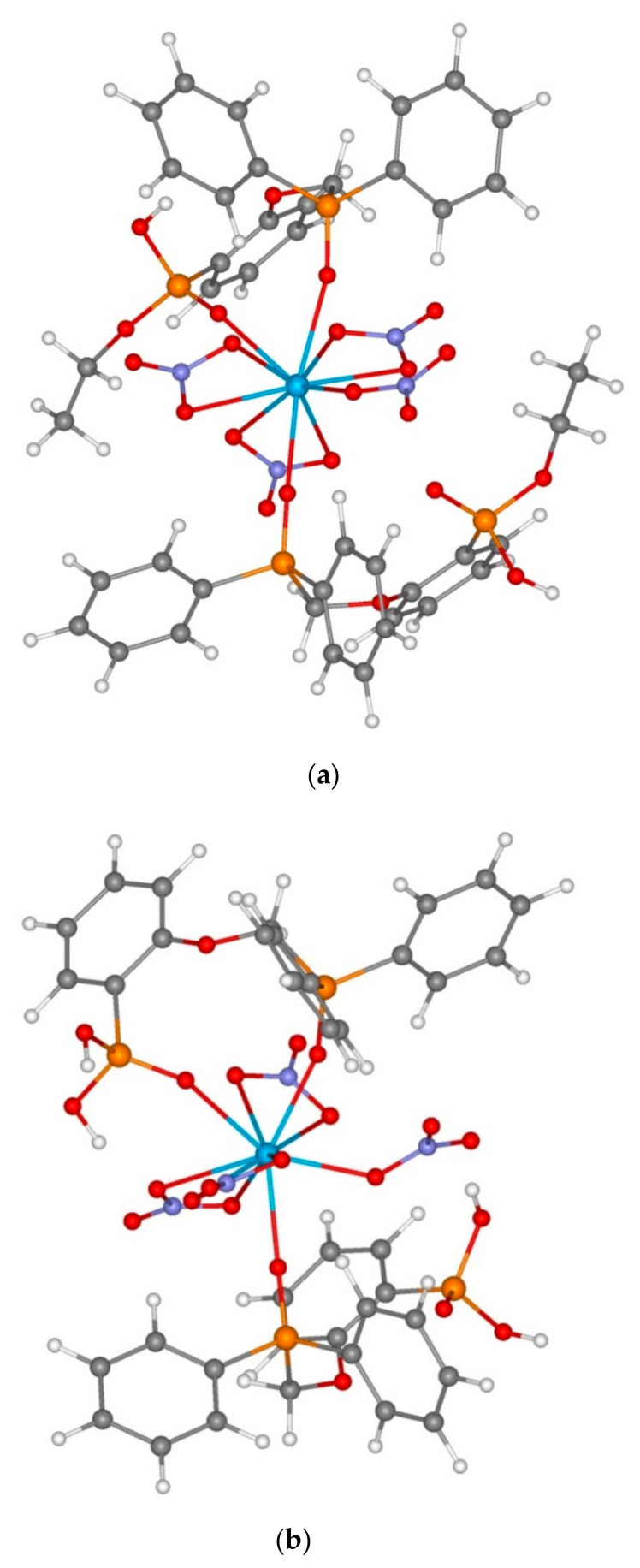
Structures of model complexes (6H)_2_Th(NO_3_)_4_ (**a**) and (7H)_2_Th(NO3_3_)_4_ (**b**).

A comparison of results of quantum chemical modelling of reagents **6** and **7** with analogous data for neutral reagents **1** and **5** allows us to draw a series of conclusions on the features of their complexation. Thus, ligands **6** and **7** comprise a part of neutral monoligand complexes of composition LTh(NO_3_)_3_ in the form of monoanions, and their bidentate coordination is analogous to that of neutral reagents **1** and **5**. For neutral complexes of composition L_2_Th(NO_3_)_4_, the coordination of two molecules of the protonated reagent is analogous of that for neutral phosphonate **5**, but not for phosphine oxide **1**. Hence, all the three phosphine acid derivatives can form complexes of similar composition. The complexation energy is remarkably different. Thus, during the formation of complexes of type LTh(NO_3_)_3_, the energy gain for reagents **6** and **7** is much greater than for their cation analogues (Table 4, nos. 2 and 3). During the formation of neutral complexes with protonated ligands, which can serve as a model of complexation in highly acidic media, the complexation energy is moderate, lower than that of ether **5** (Table 4, nos. 7 and 8). Therefore, in the range of moderate nitric acid concentrations, we can expect that reagents **6** and **7** will show a greater affinity towards thorium ions than reagent **1**; as for its high concentrations, the extraction ability can decrease to some degree, which corresponds to the classical behavior of acidic extractants and is confirmed by experimental data (Figure 12).

It is known that the extraction of lanthanides, including neodymium, with phosphine oxide **1** results in the formation of complexes of type L_2_Nd(NO_3_)_3_, as it was demonstrated in [23]. Modelling of neodymium complexes with reagents **1** and **5**–**7** was made presuming the similarity of complexation processes for all the studied ligands. All the model complexes have negative complexation energies, which indicate the general possibility of complex formation. For all the studied compounds, the oxygen atom of bridging fragment does not participate in the coordination with the metal atom since it is located at a distance longer than 4 Å. Despite all the studied reagents from complexes of the same stoichiometry, the coordination of ligands differs depending on the presence of acidic protons in the molecule. All the neodymium complexes contain three nitrate anions with the bidentate coordination, as well as one ligand with the bidentate coordination mode and one monodentate terminal organic ligand. 

**Figure 13:**
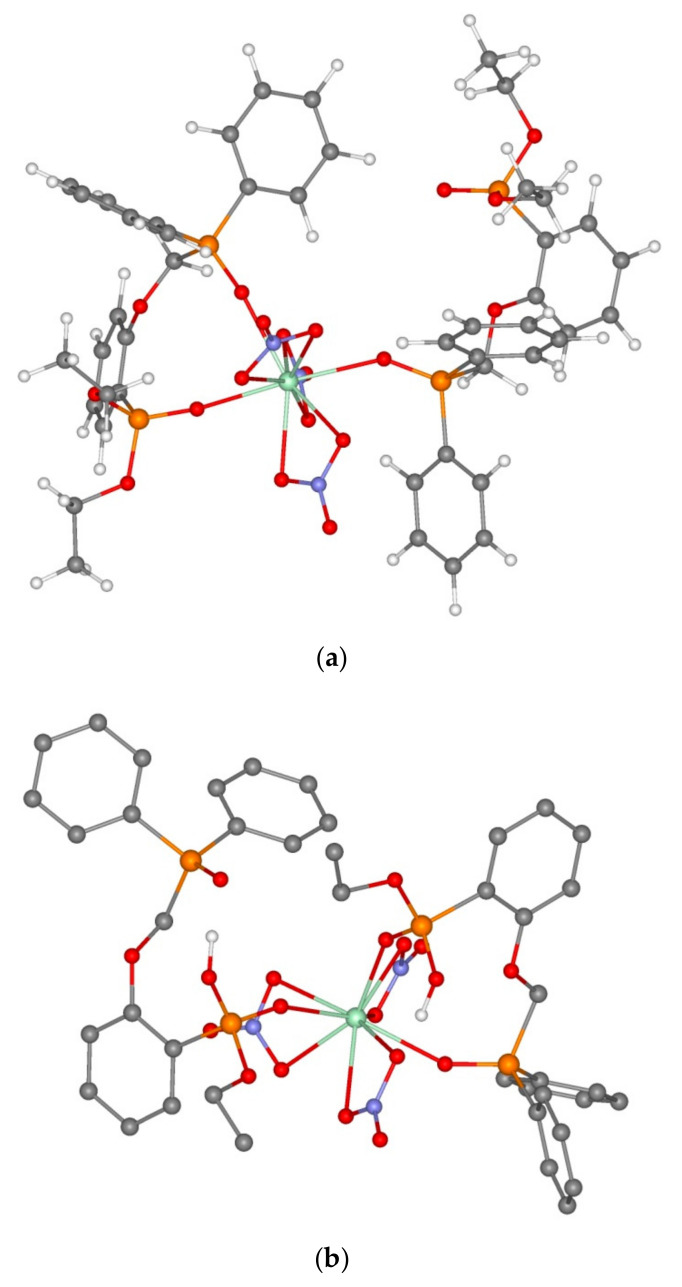
The structures of model complexes 5_2_Nd(NO_3_)_3_ (**a**) and (6H)_2_Nd(NO_3_)_3_ (**b**). For the latter complex, all the hydrogen atoms except for those involved in hydrogen bonding were omitted for clarity.

Neutral compounds **1** and **5** behaved as monodentate ligands and coordinated via the phosphine oxide group of the methylphosphinoyl fragment (Figure 13), as it was expected from its greater basicity, whereas the protonated reagents **6** and **7** shows a metal ion coordination to the phosphonate group. It occurs due to the intramolecular protonation of the more basic phosphine oxide group by the acidic proton of the reagent (Figure 12). Thus, intramolecular hydrogen bonds hold both oxygen atoms of the reagent. Moreover, the complex is additionally stabilized by intramolecular hydrogen bonds. These factors increase the complexation energy, therefore the stability of complexes drops in the sequence **1** > **7** > **6** > **5**, but the differences are small, and the complexation energies drop from **1** to **7** only by 5 kcal/mol (Table 5). This fact leads to small differences in the extraction efficiency of all the studied reagents.

Nd(NO_3_)_3_ + 2L = L_2_Nd(NO_3_)_3_ (3)

**Figure 14:**
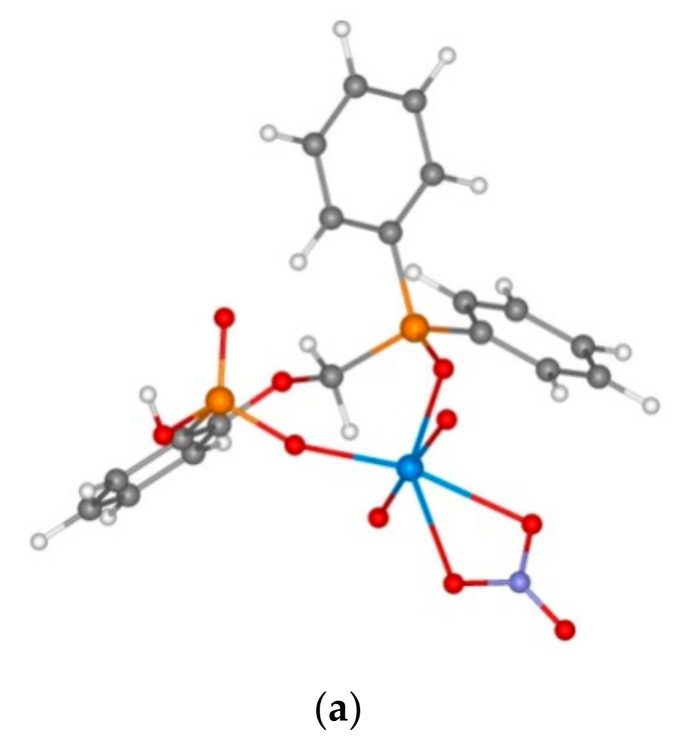

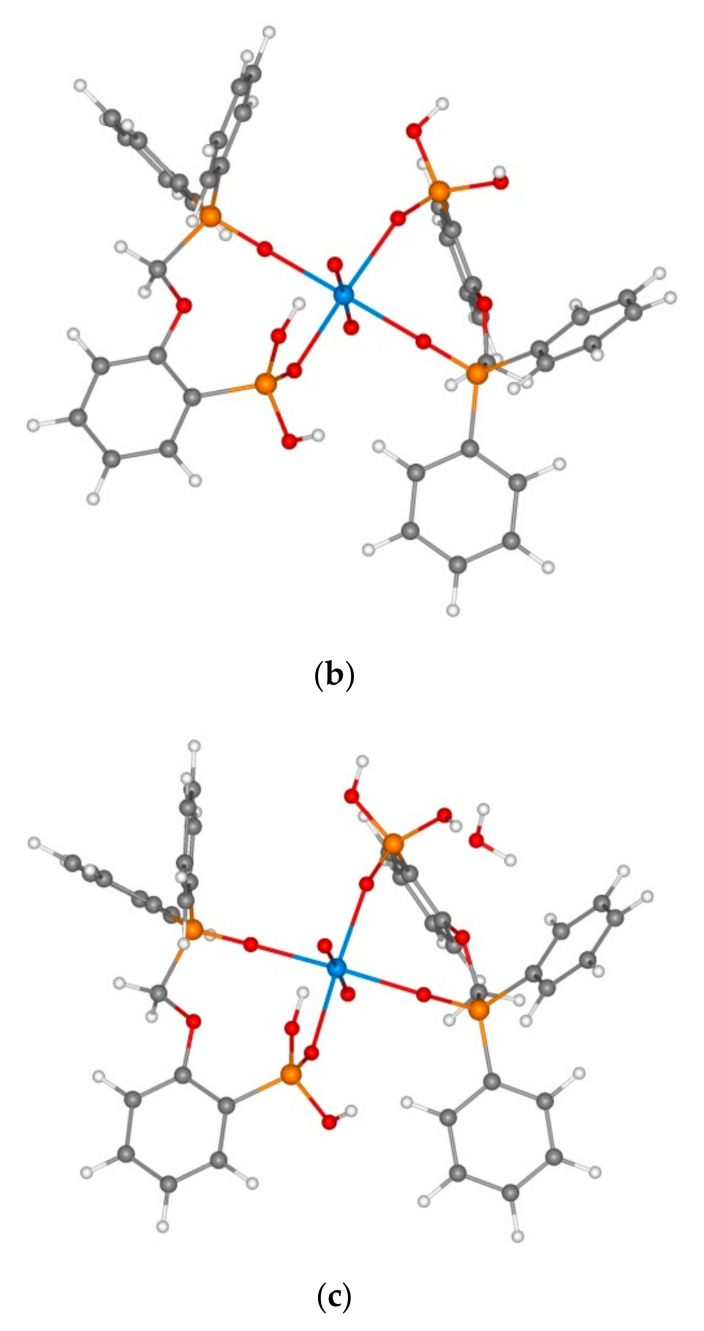
Structures of model complexes 7UO_2_(NO_3_) (**a**), (H_2_7)_2_UO_2_^2+^(**b**), (H_2_7)_2_UO_2_(H_2_O) (**c**).

**Figure 15:**
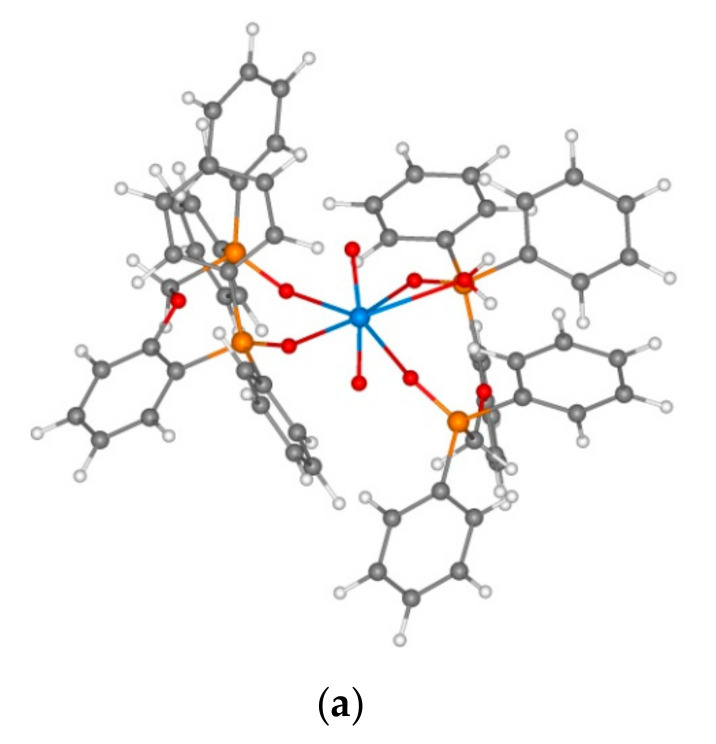

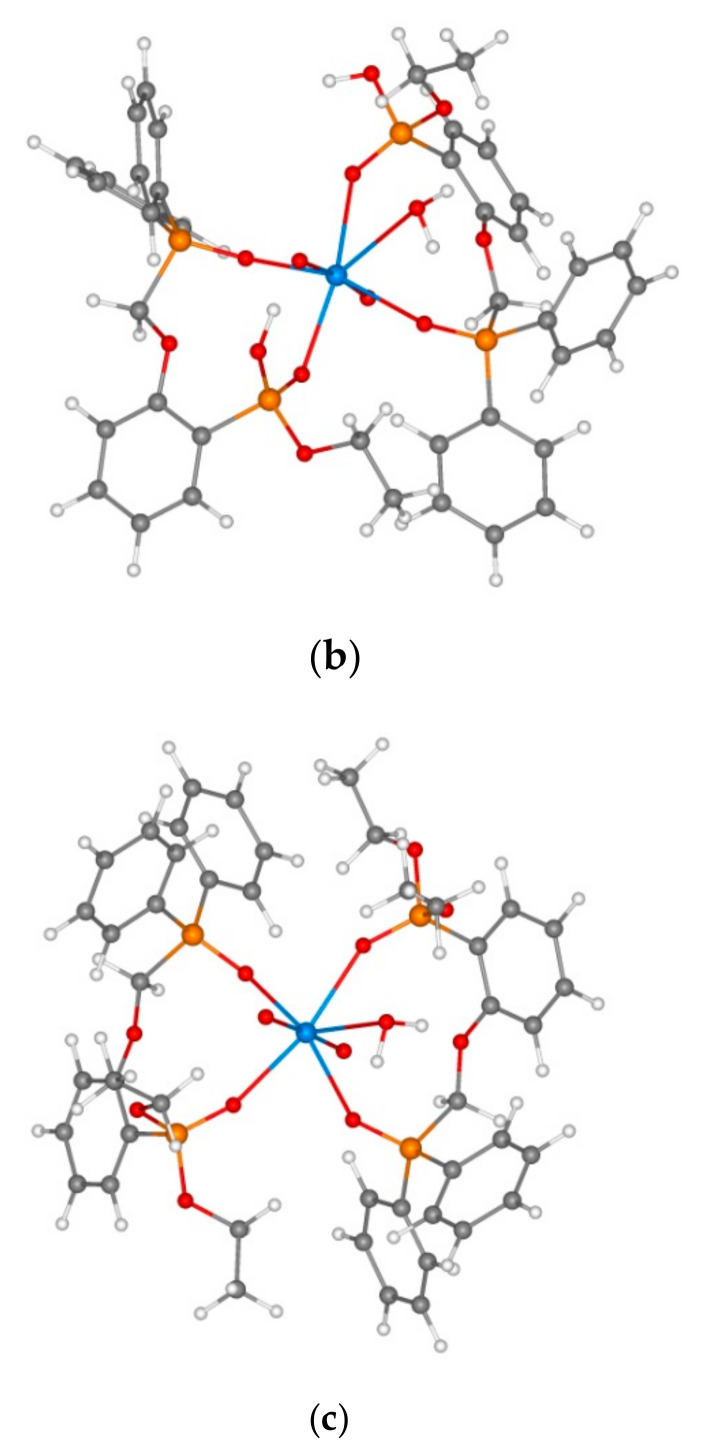
Structures of model complexes (from left to right): 1_2_UO_2_(H_2_O)(**a**), (H6)_2_UO_2_(H_2_O) (**b**)**,** and 5_2_UO_2_(H_2_O) (**c**)**.**

The difference between the extraction ability of neutral (**1** and **5**) and acidic (**6** and **7**) is most obvious for the extraction of uranium(VI). According to the literature [24], reagent **1** is used to extract complexes of both L(UO_2_)(NO_3_)_2_ and L_2_(UO_2_)(NO_3_)_2_ types. We modelled the series of complexes for all the four studied ligands. In all the studied complexes, the ligand coordination is bidentate, the oxygen atom does not participate in the coordination even in coordinatively unsaturated charged species of LUO_2_(NO_3_)^+^ type (Figure 14). The formation of model complexes of L(UO_2_)(NO_3_)^(0/+)^ type (Table 6, nos. 1–4) and L_2_(UO_2_)(NO_3_)^(0/+)^ type (Table 6, nos. 5–8) provides significant energy gains (by more than two times) for all ligands. As in the case of thorium model complexes, the formation of neutral complexes with acidic reagents **6** and **7** provides a larger gain than that with neutral compounds **1** and **5**, which agrees well with experimental data. The distance between the metal ion and the oxygen atom of phosphine oxide group is also different for these pairs of ligands: The distance for U-O is 2.32 Å for ligands **1** and **5** and 2.36 Å for ligands **6** and **7**. At the same time, the U-O distance to the oxygen atom of phosphonate group is much shorter, 2.21 Å. It may seem unexpected if one disregards the total negative charge of the ligand, which is located on the oxygen atoms. As for the metal complexation with protonated forms of acidic reagents (H_2_7 and H6), the complexation energy is much lower and therefore the U-O distance increases. However, even in this case, the distance from the metal to the oxygen atom of the phosphine oxide group is longer than that to the oxygen atom of the phosphonate P=O group. Another unusual characteristic of the complexes is the small coordination number of the metal ion. The uranyl group coordinates only to four oxygen atoms. The study of structure of possible hydrated species showed that the coordination of water molecule with the model complex of ligand **7** ((H_2_7)_2_UO_2_^2+^) occurs only due to hydrogen bonding to the OH group of the phosphonic fragment (Figure 14), while for other phosphonic reagents, the water molecule can coordinate directly to the uranium ion, thus increasing its coordination number up to **7** (Figure 15, Table 7).

UO_2_(NO_3_) + L = LUO_2_(NO_3_) (4)

UO_2_^2+^ + 2(H_n_L) = (H_n_L)_2_UO_2_^2+^ (5)

UO_2_^2+^ + 2(H_n_L) + H_2_O = (H_n_L)UO_2_(H_2_O)^2+^ (6)

The modelling results provide us with a new approach to assess the effect of podand structure on the selectivity of its binding with metal ions. The available data show that, despite the absence of coordination of the metal ion with the oxygen atom of podand, they are very close: 4.2–4.6 Å for thorium, 4.0–4.7 for neodymium, and 3.9–4.3 for uranium. The replacement of the oxygen atom by the methylene group will considerably increase the volume of the substituent and the distance between the lateral hydrocarbon chain and the metal ion, which decreases the extraction ability of reagents, as it is seen from the comparison of properties of 1,5-diphosphoryl derivatives of pentane and 3-oxapentane [30].

### 3.3. The Comparison of Extraction Ability of Compounds ***1*** and ***5***–***7*** with Commercially Available Extractants

The quantitative characteristics of the studied ligands **1**–**4** upon extraction of U(VI), Th(IV), Nd(III), and Ho(III) were compared with those of the known analogues of neutral organophosphorus compounds, i.e., tributyl phosphate (11) and diphenyldibutylcarbamoylmethylphosphine oxide (12) (Table 8), as well as acidic organophosphorus compounds, i.e., di-2-ethylhexylphosphoric acid (8), amylphosphonic acid (9), and phenylphosphonic acid (10) (Table 9). It should be noted that the extraction of actinides and lanthanides by the studied compounds **1** and **5**–**7**, and reference compounds **8**–**12** was conducted under the same experimental conditions.

The dependencies of distribution ratios (D) for actinides and lanthanides on the nitric acid concentration show a similar character for the studied compounds **1** and **5,** as well as for the analogues **11** and **12**: Ds of actinides and lanthanides increase with the HNO_3_ concentration (Table 8). An exception is the thorium(IV) extraction with a solution of ligand **1**, where D_Th(IV)_ is independent of the HNO_3_ concentration probably due to a large difference between the ligand and metal concentrations. It should be noted that the extraction efficiency of ligand **1** towards Th(IV) is much higher than that of ligand **5** and especially reference ligands **11** and **12**. The extraction efficiency of compounds **1** and **5** towards uranium(VI), as for Th(IV), is much higher than that of reference compounds **11** and **12** (Table 8). The quantitative characteristics of extraction of trivalent lanthanides are lower than those for actinides, however, D values for both neodymium and holmium are twice as large as those obtained in the extraction with analogous compounds **11** and **12** (Table 8).

Similarly, we compared the quantitative characteristics of extraction of actinides and lanthanides with acidic ligands **6** and **7** with those of reference compounds **8**–**10** (Table 9).

The distribution ratios of uranium(VI) obtained for the studied extractant **7** and reference extractant **8** in the range of HNO_3_ concentration around 0.045 mol/L are comparable (~10). It should be noted that D_U(VI)_ of extractant **7** is much higher than D_U(VI)_ of reference extractants **9** and **10**. For uranium(VI) extraction, D_U(VI)_ = 1.83 in the range of C_HNO3_ = 0.045 mol/L for the solution of ligand **6**, which is lower than that for reference compound **8**, but higher than that for reference compounds **9** and **10** (Table 9). The extraction of uranium(VI) at C_HNO3_ = 3.86 mol/L with solutions of studied ligands **6** and **7** is more efficient than that with reference extractants **8**–**10** (Table 9).

Solutions of ligands **6** and **7** and reference compounds **8**–**10** quantitatively extract Th(IV) at C_HNO3_ = 0.045 mol/L. However, the extraction ability of reference compounds **8**–**10** towards Th(IV) at C_HNO3_ = 3.86 mol/L is much lower than that of studied compounds **6** and **7**, which provide quantitative Th(IV) extraction at the same HNO_3_ concentration.

The extraction ability of studied acidic ligands **6** and **7**, and reference compounds **8**–**10** towards trivalent lanthanides is comparable. An exception is ligand **7** whose extraction ability is more than twice as high as the rest of the ligands. It should be noted that neodymium and holmium have relatively high distribution ratios at C_HNO3_ = 0.045 mol/L. 

Thus, studied compounds **1** and **5**–**7** are promising extractants for extracting and separating actinides and lanthanides. The difference in the D values for the studied elements provides a possibility for their efficient extraction separation by processing technical wastes of various origins.

## 4. Conclusions

In this work, we compared the extraction ability of 2-((diphenylphosphoryl)methoxy)phenyl)diphenyl)phosphine oxide **1** and its analogues **5**–**7,** in which the ArP(O)Ph_2_ group of phosphine oxide type was replaced by a phosphonic fragment. Quantum-chemical modelling of the structures of compounds **1** and **5**–**7** was conducted. Models were confirmed by the results of X-ray diffraction analysis. The features of extraction of nitric acid and U(VI), Th(IV), Nd(III), and Ho(III) from nitric acid solutions into 1,2-dichloroethane were studied. The extraction ability of compounds **1** and **5**–**7** towards U(VI), Th(IV), Nd(III), and Ho(III) was compared with reference acidic extractants, such as di-(2-ethylhexyl)phosphoric acid, amylphosphonic and diphenylphosphinic acids, as well as neutral extractants (tributyl phosphate and N,N-dibutyl-2-(diphenylphosphoryl)acetamide).

## Figures and Tables

**Table 1:** Crystallographic data, parameters of experiment, and refinement for structures **6** and **7.**

Parameter	6	7
Molecular formula	C_21_H_22_O_5_P_2_	C_19_H_18_O_5_P_2_
*M*	416.32	388.27
Syngony, space group, *Z*	Monoclinic, *P*2_1_/*n*, 4	Monoclinic, *P*2_1_/*c*, 4
*a*, Å	8.0671(13)	13.0212 (3)
*b,* Å	18.393(4)	11.2464 (3)
*c,* Å	14.410(3)	12.5224 (3)
β, degrees	92.309(8)	105.213 (1)
*V*, Å^3^	2136.5(7)	1769.54 (8)
*D_x_*, g/cm^3^	1.294	1.457
Radiation, λ, Å	Mo*K*_α_, 0.71073	Mo*K*_α_, 0.71073
*T*, K	100(2)	100 (2)
Size of crystal, mm	0.40 × 0.10 × 0.10	0.26 × 0.20 × 0.18
θ_max_, degrees	27.5	30
*h*, *k*, *l* range	−10 ≤ h ≤ 6, −23 ≤ k ≤ 20, −17 ≤ l ≤ 18	−18 ≤ h ≤ 18, −15 ≤ k ≤ 15, −17 ≤ l ≤ 17
Number of reflections: Measured/independent, *R*_int_	14159/4729, 0.0759	46482/5118, 0.0325
Number of refined parameters	257	243
*R*(*F*), *wR*(*F*^2^) [*I* > 2σ(*I*)]	0.0499, 0.0978	0.0312, 0.0794
*R*(*F*), *wR*(*F*^2^) [whole array]	0.0960, 0.1153	0.0377, 0.0833
*S*	0.988	1.052
Δρ_max_, Δρ_min_, e/Å^3^	0.358, −0.418	0.472, −0.268

**Table 2:** Hydrogen bonds in structures **6** and **7.**

*D*–H…*A*	*D*–H, Å	H…*A*, Å	*D*…*A*, Å	*D*–H…*A*, Degrees	Symmetry Operation for *A*
**6**					
O(2)-H(2)...O(5)	0.78(3)	1.78(3)	2.562(2)	177(4)	1 + *x*, *y*, *z*
C(36)-H(36A)...O(2)	0.95	2.34	3.196(3)	149	
**7**					
O(2)-H(2)...O(1)	0.80(3)	1.77(3)	2.5725(13)	176(3)	2-*x*, 1-*y*, 1-*z*
O(3)-H(3)...O(5)	0.79(3)	1.74(3)	2.5190(12)	169(3)	

**Table 3:** Charges at the oxygen atoms of phosphoryl groups estimated by the Mulliken analysis and parameters (distances in Å and angles) of intramolecular hydrogen bonds for podands **1** and **5–7.**

	1	5	6	7
q O=PAr_3_	−0.4801	−0.4735	−0.5100	−0.5182
q O=P(R_2_)CH2	−0.4701	−0.4698	−0.4901	−0.4816
H…O=P	-	-	1.647 (164.167)	1.636 (169.931)

**Table 4:** Complexation energies (kcal/mol, Equations (1) and (2)) and interatomic distances (Å) for the model complexes of ligands **1** and **5**–**7** with thorium nitrate.

No.	Complexes	ΔE, kcal/mol (Equation (1) or (2))	Th-O=PCH_2_, Å	Th-O=P(OEt)	Th-O=PPh_2_	Th…O
1	1Th(NO_3_)_3_^+^	−126.63	2.338		2.307	4.236
2	(7H)Th(NO_3_)_3_	−211.43	2.368	2.216		4.309
3	6Th(NO_3_)_3_	−211.18	2.369	2.204		4.298
4	5Th(NO_3_)_3_^+^	−119.77	2.330	2.354		4.291
5	1_2_Th(NO_3_)_3_^+^	−180.12	2.417		2.436	4.388/4.385
6	1_2_Th(NO_3_)_4_	−60.40	2.458/2.500		2.675/2.652	4.582/4.787
7	(7H_2_)_2_Th(NO_3_)_4_	−54.92	2.439/2.398		2.465/4.987	4.656/5.419
8	(6H)_2_Th(NO_3_)_4_	−42.43	2.535/2.429		2.519/3.796	4.669/5.073
9	5_2_Th(NO_3_)_4_	−56.12	2.524/2.445	2.519/3.811		4.613/5.116

**Table 5:** The complexation energies (kcal/mol, Equation (3)) and interatomic distances (Å) for the model complexes of ligands **1** and **5** with neodymium nitrate.

No.	Complexes	ΔE, kcal/mol (Equation (3))	Nd-O=PCH_2_, Å	Nd-O=P(OEt), Å	Nd-O=PPh_2_, Å	Nd…O, Å
1	**1_2_Nd(NO_3_)_3_**	−72.48	2.528/2.467		2.408/5.938	4.296/5.645
2	**(7H)_2_Nd(NO_3_)_3_**	−69.29	2.446/3.913	2.384/2.642		4.715/5.787
3	**(6H)_2_Nd(NO_3_)_3_**	−69.05	2.457/5.294	2.490/2.445		4.013/5.689
4	**5_2_Nd(NO_3_)_3_**	−63.04	2.504/2.486	2.453/6.794		4.474/5.782

**Table 6:** Complexation energies (kcal/mol, Equations (4)–(6)) and interatomic distances (Å) for model complexes of ligands **1** and **5** with uranyl nitrate.

No.	Complexes	ΔE, kcal/mol (Equations (4)–(6))	U-O=PCH_2_, Å	U-O=P(OEt), Å	U-O=PPh_2_, Å	U…O, Å
1	1UO_2_(NO_3_)^+^	−134.91	2.325		2.285	3.852
2	7UO_2_(NO_3_)	−218.23	2.354	2.207		3.563
3	6UO_2_(NO_3_)	−216.38	2.368	2.215		3.383
4	5UO_2_(NO_3_)^+^	−128.83	2.322	2.303		3.534
5	1_2_UO_2_^2+^	−393.01	2.337/2.337		2.302/2.302	4.188/4.187
6	(7H)_2_UO_2_	−586.22	2.399/2.356	2.222/2.233		4.057/3.800
7	6UO_2_	−579.00	2.415/2.366	2.244/2.244		4.197/3.754
8	5_2_UO_2_^2+^	−377.95	2.319/2.321	2.325/2.327		4.127/4.111
9	(7H_2_)_2_UO_2_^2+^	−357.87	2.320/2.340	2.290/2.297		3.986/3.830
10	(6H)_2_UO_2_^2+^	−359.77	2.314/2.319	2.323/2.336		4.120/3.722
11	1_2_UO_2_(H_2_O)^2+^	−395.09	2.350/2.445		2.325/2.346	4.321/3.625
12	(7H_2_)_2_UO_2_(H_2_O)^2+^	−376.28	2.311/2.316	2.294/2.293		4.006/4.878
13	(6H)_2_UO_2_(H_2_O)^2+^	−591.61	2.350/2.485	2.418/2.484		4.118/3.644
14	5_2_UO_2_(H_2_O)^2+^	−596.81	2.348/2.476	2.454/2.448		4.341/3.642

**Table 7:** Interatomic distances (Å) and valence angles in water molecules of model complexes of ligands **1** and **5** of type L_2_UO_2_(H_2_O)^2+.^

	1	5	6	7
U-OH_2_, Å	2.768	2.855	2.840	4.878
HO-H…O, Å	-	1.968/2.103	1.936	1.519
HO-H…O, deg.	-	150.5/121.2	131.59	176.38

**Table 8:** Comparison of distribution ratios for U(VI), Th(IV), Nd(III), and Ho(III) on the extraction with the 0.01 mol/L solution of studied neutral compounds **1** and **5** and neutral organophosphorus compounds **11** and **12**.

**Compound**	**U(VI)**		**Th(IV)**		**Nd(III)**		**Ho(III)**	
	**C_HNO3_, mol/L**							
	0.045	3.86	0.045	3.86	0.045	3.86	0.045	3.86
	**Distribution Ratio (D)**							
**1**	0.17	10.3	53	53	0.09	0.7	0.11	0.82
**5**	2.24	12.3	0.040	16	0.20	0.79	0.25	0.85
**11**	0.019	0.38	0	0.19	0	0.35	0.04	0.42
**12**	0.05	0.60	0.030	0.20	0	0.33	0	0.38

**Table 9:** The comparison of distribution ratios for U(VI), Th(IV), Nd(III), and Ho(III) upon extraction with 0.01 mol/L solutions of studied acidic compounds **6** and **7** and of analogous acidic organophosphorus compounds **8–10.**

**Compound**	**U(VI)**		**Th(IV)**		**Nd(III)**		**Ho(III)**	
	**C_HNO3_, mol/L**							
	0.045	3.86	0.045	3.86	0.045	3.86	0.045	3.86
	**Distribution Ratio (D)**							
**6**	1.83	4.68	50	51	0.25	0.82	0.22	1.37
**7**	9.88	10.8	95	70	2.28	2.69	2.1	3.86
**8**	10	0.62	74	0.056	0.11	1.08	0.24	1.68
**9**	1.08	0.49	52	0.05	0.24	0.71	0.06	0.73
**10**	0.023	0.47	53	0	0.13	0.49	0.07	1.34

## Data Availability

Not available.

## Supplementary Materials

The following materials are available online. Table S1: Selected bond distances (d) and bond angles (ω) in structures **6** and **7**.

## Sample Availability

Samples of the compounds (**1**, **5**–**7**) are available from the Dmitry Baulin and Vladimir Baulin.

## References

[1] Paulick H., Machacek E. (2017). The global rare earth element exploration boom: An analysis of resources outside of China and discussion of development perspectives. Resour. Policy.

[2] Hill C., Moyer B.A. (2010). Overview of Recent Advances in An(III)/Ln(III) Separations. Ion Exchange and Solvent Extraction: A Series of Advances.

[3] Tachimori S., Morita Y. (2010). Overview of solvent extraction chemistry for reprocessing. Ion Exch. Solvent Extr..

[4] Leoncini A., Huskens J., Verboom W. (2017). Ligands for f-element extraction used in the nuclear fuel cycle. Chem. Soc. Rev..

[5] Safiulina A.M., Anan’ev A.V., Lizunov A.V., Tuiza M., Logunov M.V., Dvoeglazov K.N. (2020). Experimental Modeling of Technetium(VII) Recoveryfrom Raffinates after Extractive Reprocessing of Spent Nuclear Fuel. Russ. J. Inorg. Chem..

[6] Batchu N.K., Li Z., Verbelen B., Binnemans K. (2021). Structural effects of neutral organophosphorus extractants on solvent extraction of rare-earth elements from aqueous and non-aqueous nitrate solutions. Sep. Purif. Technol..

[7] Flett D.S. (2005). Solvent extraction in hydrometallurgy: The role of organophosphorus extractants. J. Organomet. Chem..

[8] Rozen A.M., Krupnov B.V. (1996). Dependence of the extraction ability of organic compounds on their structure. Russ. Chem. Rev..

[9] Sharova E.V., Artyushin O.I., Odinets I.L. (2014). Synthetic routes to carbamoylmethylphosphoryl compounds—Extractants for the processing of spent nuclear fuels. Russ. Chem. Rev..

[10] Matveev P., Mohapatra P.K., Kalmykov S.N., Petrov V. (2020). Solvent extraction systems for mutual separation of Am(III) and Cm(III) from nitric acid solutions. A review of recent state-of-the-art. Solvent Extr. Ion Exch..

[11] Jensen M., Chiarizia R., Ulicki J.S., Spindler B.D., Murphy D., Hossain M.M., Roca-Sabio A., Blas A., Rodríguez-Blas T. (2015). Solvent Extraction Separation of Trivalent Americium from Curium and the Lanthanides. Solvent Extr. Ion Exch..

[12] Mahanty B., Mohapatra P.K., Leoncini A., Huskens J., Verboom W. (2019). Evaluation of three novel benzene-centered tripodal diglycolamide ligands for the pertraction of americium(III) through flat sheet membranes for nuclear waste remediation applications. Sep. Purif. Technol..

[13] Sasaki Y., Umetani S. (2006). Comparison of Four Bidentate Phosphoric and Diamide Compounds for the Extractability of Actinides. J. Nucl. Sci. Technol..

[14] Dam H.H., Reinhoudt D.N., Verboom W. (2007). Multicoordinate ligands for actinide/lanthanide separations. Chem. Soc. Rev..

[15] Tatarinov D.A., Mironov V.F., Kostin A.A., Nemtarev A.V., Baronova T.A., Buzykin B.I., Elistratova Y.G. (2011). A New Versatile Synthesis of Functionalized Phosphine Oxides—Efficient Ligands for Rare-Earth Metals Extraction. Phosphorus Sulfur Silicon Relat. Elem..

[16] Swinburne A.N., Steed J.W., Gale P.A., Steed J.W. (2012). Podands. Supramolecular Chemistry.

[17] Werner E.J., Biros S.M. (2019). Supramolecular ligands for the extraction of lanthanide and actinide ions. Org. Chem. Front..

[18] Borisova N.E., Safiullina A.M., Lizunov A.V., Semenov A.A., Grigor’ev M.S., Reshetova M.D., Litvinov I.A., Tatarinov D.A., Mironov V.F. (2019). Metal-Promoted Extraction Deprotonation of Bidentate Organophosphoric Reagents: Recovery of Uranium, Thorium, and Lanthanides. Russ. J. Inorg. Chem..

[19] Mohapatra P.K., Kandwal P., Iqbal M., Huskens J., Murali M.S., Verboom W. (2013). A novel CMPO-functionalized task specific ionic liquid: Synthesis, extraction and spectroscopic investigations of actinide and lanthanide complexes. Dalton Trans..

[20] Charpentier C., Salaam J., Lecointre A., Jeannin O., Nonat A., Charbonnière L.J. (2019). Phosphonated Podand Type Ligand for the Complexation of Lanthanide CationsPhosphonated Podand Type Ligand for the Complexation of Lanthanide Cations: Phosphonated Podand Type Ligand for the Complexation of Lanthanide Cations. Eur. J. Inorg. Chem..

[21] Turanov A.N., Karandashev V.K., Baulin V.E. (1996). Extraction of Metal Chloride Complexes by Phosphoryl-Containing Podands. Solvent Extr. Ion Exch..

[22] Turanov A.N., Karandashev V.K., Baulin V.E. (1996). Extraction of Metal Chloride Complexes by Phosphoryl-Containing Azapodands. Solvent Extr. Ion Exch..

[23] Turanov A.N., Karandashev V.K., Baulin V.E. (1999). Extraction of Rare-Earth Elements from Nitric Solutions by Phosphoryl-Containing Podands. Solvent Extr. Ion Exch..

[24] Turanov A.N., Karandashev V.K., Fedoseev A.M., Rodygina N.I., Baulin V.E. (2005). Effect of the Structure of o-Methyleneoxyphenyldiphosphine Dioxides on Their Extractive Power and Selectivity in Nitric Acid Solutions. Radiochemistry.

[25] Turanov A.N., Karandashev V.K., Yarkevich A.N., Baulin V.E. (2009). Extraction of U(VI) and Th(IV) from nitric acid solutions with tetraaryl-substituted (o-phenyleneoxymethylene)diphosphine dioxides. Radiochemistry.

[26] Turanov A.N., Karandashev V.K., Baulin V.E., Yarkevich A.N., Safronova Z.V. (2009). Extraction of Lanthanides(III) from Aqueous Nitrate Media with Tetra-(p-tolyl)[(o-Phenylene)Oxymethylene] Diphosphine Dioxide. Solvent Extr. Ion Exch..

[27] Demin S.V., Zhilov V.I., Nefedov S.E., Baulin V.E., Tsivadze A.Y. (2012). Extraction of rare earth elements with 1-(diphenylphosphorylmethoxy)-2-diphenylphosphoryl-4-ethylbenzene with the use of 1,1,7-trihydrododecafluoroheptanol as a solvent. Russ. J. Inorg. Chem..

[28] Polyakova I.N., Baulin V.E., Ivanova I.S., Pyatova E.N., Sergienko V.S., Tsivadze A.Y. (2015). Crystal and molecular structure of the coordination compounds of Er3+ with 1-(methoxydiphenylphosphoryl)-2-diphenylphosphorylbenzene [ErL21(NO_3_)_2_]2[Er(NO_3_)_2_(H_2_O)5]0.333(NO_3_)2.333 2.833H2O and its ethyl substituted derivative [ErL22(NO_3_)2][Er(NO_3_)5]0.5 · 0.5H_2_O. Crystallogr. Rep..

[29] Turanov A.N., Karandashev V.K., Baulin V.E., Kalashnikova I.P., Kirillov E.V., Kirillov S.V., Rychkov V.N., Tsivadze A.Y. (2016). Extraction of rare earths and scandium by 2-phosphorylphenoxyacetic acid amides in the presence of ionic liquids. Russ. J. Inorg. Chem..

[30] Safiulina A.M., Matveeva A.G., Ivanets D.V., Kudryavtsev E.M., Grigor’ev M.S., Baulin V.E., Tsivadze A.Y. (2015). Phosphoryl-containing acidic podands as extractants for recovery of f-elements: Synthesis and comparison of podands different in polyether chain length and structure. Russ. Chem. Bull..

[31] Safiulina A.M., Matveeva A.G., Ivanets D.V., Kudryavtsev E.M., Baulin V.E., Tsivadze A.Y. (2015). Phosphoryl-containing acidic podands as extractants for recovery of f-elements: Synthesis and comparison of podands different in terminal group structure. Russ. Chem. Bull..

[32] Timofeeva G.I., Matveeva A.G., Safiulina A.M., Ivanets D.V., Kudryavtsev E.M., Baulin V.E., Tsivadze A.Y. (2015). Phosphoryl-containing acidic podands as extractants for recovery of f-elements: Dependence of the degree of association of podands on the nature of substituent and concentration in water–methanol solutions. Russ. Chem. Bull..

[33] Laikov D.N. (2005). A new class of atomic basis functions for accurate electronic structure calculations of molecules. Chem. Phys. Lett..

[34] Laikov D.N. (1997). Fast evaluation of density functional exchange-correlation terms using the expansion of the electron density in auxiliary basis sets. Chem. Phys. Lett..

[35] Perdew J.P., Burke K., Ernzerhof M. (1997). Generalized Gradient Approximation Made Simple. Phys. Rev. Lett..

[36] Bruker (2006). APEX2.

[37] Sheldrick G.M. (2008). A short history of SHELX. Acta Crystallogr. Sect. A Found. Crystallogr..

[38] Sheldrick G.M. (2015). Crystal structure refinement with SHELXL. Acta Crystallogr. Sect. C Struct. Chem..

[39] Savvin S.B. (1966). Arsenazo III.

[40] Hamed M.M., Aglan R.F. (2019). Removal of Arsenazo-III from liquid radioactive waste by cloud point extraction. J. Radioanal. Nucl. Chem..

