# Supplementary material for: Design of Extractants for F-Block Elements in a Series of (2-(Diphenylphosphoryl)methoxyphenyl)diphenylphosphine Oxide Derivatives: Synthesis, Quantum-Chemical, and Extraction Studies
Source: Molecules. 2021 Apr 12;26(8):2217. doi: 10.3390/molecules26082217 (PMC8069430; doi:10.3390/molecules26082217)
Supplement: Supplementary file 1 [file molecules-26-02217-s001.pdf]

Supporting information

Design of Extractants for F-Block Elements in a Series of (2-(Diphenylphosphoryl)methoxyphenyl)diphenylphosphine Oxide Derivatives: Synthesis, Quantum-Chemical and Extraction Studies

Alfiya Safiulina[1], Nataliya Borisova[2], Mikhail Grigoriev[3], Dmitriy Baulin[3,*], Vladimir Baulin[4] and Aslan Tsivadze[3]

[1] Joint Stock Company A. A. Bochvar High-technology Research Institute of Inorganic Materials, ul. Rogova 5a, Moscow, 123098 Russia
[2] Moscow State University, Leninskie Gory 1, Moscow, 119991 Russia
[3] A. N. Frumkin Institute of Physical Chemistry and Electrochemistry, Russian Academy of Sciences, Leninsky prospect 31, building 4, Moscow, 119071 Russia
[4] Institute of Physiologically Active Substances, Russian Academy of Sciences, Severnyi proezd 1, Chernogolovka, Moscow Region, 142432 Russia,

* Correspondence: badmitriy@gmail.com; Tel.: +7-903-724-36-60

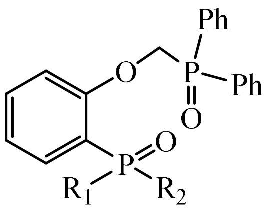

6-7

6, R_1=OEt, R_2=OH

7, R_1=OH, R_2=OH

Table S1. Selected bond distances (*d*) and bond angles (ω) in structures **6** and **7**

Bond	*d*, Å	Angle	ω, degrees
		**6**	
P(1)-O(1)	1.4827(18)	O(1)-P(1)-O(2)	113.26(10)
P(1)-O(2)	1.565(2)	O(1)-P(1)-O(3)	114.94(10)
P(1)-O(3)	1.5923(19)	O(1)-P(1)-C(11)	110.93(11)
P(1)-C(11)	1.798(3)	O(2)-P(1)-O(3)	104.87(10)
		O(2)-P(1)-C(11)	110.37(11)
		O(3)-P(1)-C(11)	101.74(10)
P(2)-O(5)	1.5111(17)	O(5)-P(2)-C(1)	106.68(10)
P(2)-C(1)	1.832(3)	O(5)-P(2)-C(21)	115.51(10)
P(2)-C(21)	1.816(3)	O(5)-P(2)-C(31)	111.60(10)
P(2)-C(31)	1.801(2)	C(1)-P(2)-C(21)	106.66(12)
		C(1)-P(2)-C(31)	108.84(11)
		C(21)-P(2)-C(31)	107.29(11)
		**7**	
P(1)-O(1)	1.4969(9)	O(1)-P(1)-O(2)	113.23(5)
P(1)-O(2)	1.5550(9)	O(1)-P(1)-O(3)	114.23(5)
P(1)-O(3)	1.5481(9)	O(1)-P(1)-C(11)	111.50(5)
P(1)-C(11)	1.8002(11)	O(2)-P(1)-O(3)	102.56(5)

		O(2)-P(1)-C(11)	105.51(5)
		O(3)-P(1)-C(11)	109.11(5)
P(2)-O(5)	1.5021(9)	O(5)-P(2)-C(1)	109.58(5)
P(2)-C(1)	1.8219(12)	O(5)-P(2)-C(21)	111.61(5)
P(2)-C(21)	1.7901(11)	O(5)-P(2)-C(31)	112.97(5)
P(2)-C(31)	1.7926(12)	C(1)-P(2)-C(21)	106.09(5)
		C(1)-P(2)-C(31)	108.08(5)
		C(21)-P(2)-C(31)	108.21(5)
